# Success4Life Youth Empowerment Pilot for Promoting Well-Being in University Students: A Qualitative Study

**DOI:** 10.7759/cureus.72858

**Published:** 2024-11-01

**Authors:** Sajita Setia, Michelle Tichy

**Affiliations:** 1 Health and Medical Education, Transform Medical Communications Limited, Auckland, NZL; 2 Social-Emotional Learning, Transforming Life LLC, Wilmington, USA; 3 Psychology/Educational Psychology, Alfred University, New York, USA

**Keywords:** adolescents, mental health, mental well-being, primary prevention, qualitative study, success4life, success4life youth empowerment, universal prevention, well-being, young adults

## Abstract

Background and aim

The Success4Life (S4L) program is a web-based, holistic social-emotional learning (SEL) initiative aimed at improving the mental wellness of adolescents and young adults. It adopts a coaching student-led active learning methodology that integrates hands-on experiences with positive psychology along with inquiry-based learning (IBL) and project-based learning. The objective of this study was to examine and understand the experiences of the participants with the S4L program when conducted per the published protocol.

Methods

This primarily qualitative exploratory study adopted a mixed-methods study design based on a previously published protocol. This preliminary study examined the experiences of the first cohort of students in the pilot phase at a mid-Atlantic university in the United States.

Results

A total of 10 students were approached, and four expressed interest in participating and completed the informed consent form. Three out of four participants completed the full program. The thematic analysis from the focus group interview of the three participants who successfully completed the S4L program revealed several critical findings. The participants appreciated the program's effectiveness in enhancing self-reflection and self-awareness and reducing stress through improved social interactions, enhanced self-care, and positive thinking. Practical skills such as time management and mindful social media usage were considerably enhanced, contributing to better sleep patterns and increased personal productivity. However, feedback from the participants highlighted the need for incorporating more interactive and peer-based learning methods.

Conclusions

The study suggests integrating more real-time discussions and peer interactions to enhance the learning experience in synchronous virtual settings. Overall, the S4L program demonstrates substantial potential in fostering significant improvements in mental health and well-being among the youth, with recommendations for enhancing peer-based interactive elements in future iterations.

## Introduction

Rising rates of mental illness, problematic behaviors, and social challenges have prompted both scientists and policymakers to seek effective interventions [1,2]. Social-emotional learning (SEL) has emerged as a focal point in recent literature, offering a promising approach to addressing these societal issues [3]. However, the broad scope that makes SEL appealing is the inclusion of all social and emotional skill-building forms, which also presents challenges in its conceptualization and implementation [1]. Ongoing debates concern which specific skills constitute SEL, the distinctions between various programs and approaches, and their comparative effectiveness [1]. These conceptual ambiguities hinder implementation efforts, with studies indicating that among various implementation factors, the quality of delivery, number of learners in a group, and duration of implementation significantly impact outcomes [2,4,5]. Therefore, high-quality implementation and comprehensive assessment across multiple dimensions are essential for achieving positive results with SEL programs.

Additionally, the existing SEL programs do not cover preventive aspects for drug use or the compulsive use of smartphones and social media [6,7]. In schools and colleges, prevention programs often narrowly focus on specific issues such as substance abuse, and similarly, limited approaches are taken to promote the mindful use of social media [6-8]. Yet, the significant global burden of substance abuse disorders and the problematic use of social media highlights the need for interventions that extend beyond improved diagnosis and treatment to include better and more widely accessible prevention programs [7,9-11]. Research on the interrelationship between drug abuse and social stress has primarily focused on stress exposure during emerging adulthood and adolescence [12]. Adolescence and emerging adulthood are the phases of life with heightened reward sensitivity but are also critical periods when earlier life experiences are expressed [13]. Therefore, it is crucial to design a holistic SEL program that also incorporates skill sets for the conscious and safe use of technology and substance use prevention.

The Success4Life (S4L) Youth Empowerment program is a 10-week, web-based, positive psychology intervention and a holistic SEL designed to improve the mental wellness of adolescents and young adults. It utilizes a coaching methodology that incorporates hands-on experiences in positive psychology, focusing on inquiry-based learning (IBL) and project-based learning [14,15]. The lessons cover a wide range of positive psychology elements, including stress management through adaptive coping and mindfulness-based stress reduction, self-compassion, goal-setting, learned optimism, resilience, and the awareness of the dangers of substance abuse and the mindful use of technology and social media. Participants engage in student-directed activities through weekly workshops delivered through learning and educational techniques that foster reflection and problem-solving in real-world contexts. The curriculum emphasizes inquiry-based and project-based learning, allowing students to apply psychological concepts to real-world situations through reflective journaling and structured projects. A comprehensive infographic overview of the theme, workshop-specific details, and methodologies of the S4L program is described in Appendix 1 of the published protocol by Setia et al. [15]. Qualitative methods are quite effective for mental health-related research in providing profound insights and a comprehensive understanding of the participants' experiences. These methods also enable the exploration of under-researched topics and the design and development of new approaches [16]. The objective of this study was to examine and understand the experiences of the participants from the first cohort of the S4L program at a small mid-Atlantic university conducted per the published protocol. This includes a detailed assessment of the program's impact on the participants' mental well-being and personal development, including the interventions and activities in week 9, which featured learned optimism project presentations. This initial feedback is crucial as it provides firsthand accounts of the program's impact and effectiveness directly from those it is designed to help. These insights will inform the refinement of the program's content, structure, and delivery methods, ensuring that subsequent iterations are more closely aligned with the needs and experiences of future participants [15].

## Materials and methods

The first cohort of the S4L pilot phase proceeded over 10 weeks from March 2024 to May 2024. The coaching incorporated synchronous and asynchronous learning modalities to optimize engagement and educational outcomes. Each weekly session lasted between 60 and 90 minutes and blended real-time synchronous interactive learning through videoconferencing via Zoom with asynchronous learning via a learning management system (LMS) that hosts video modules for refreshers and tracked assignment submissions while ensuring data privacy. Table 1 summarizes the topics and lessons for each workshop and their learning objectives. Further details for each workshop are available in our previous publication of the S4L program and implementation protocol [15].

**Table 1:** Course curriculum for Success4Life Youth Empowerment. All workshops utilize several inquiry- or project-based learning techniques under each lesson.

Workshop	Key lessons covered	Learning objectives
Week 1: understanding the most complex yet extremely powerful resource	Understanding our mind. Mind-body problem. The power of our mind	Impart awareness and appreciation of the innate potential in each of us, which can be harnessed by learning to control and shape our mind and thought process
Week 2: unlocking our potential	Understanding our unconscious mind. Reprogramming our unconscious mind. How to apply knowledge in real-life situations	Impart understanding related to automatic, unconscious thought processes
Week 3: self-transformation	What is self-transformation? Relationship between self-transformation and happiness, abundance, and success. Transforming the old self into a new empowered self	Explore how expanding consciousness could help achieve self-transformation with a deeper fulfillment and purpose in life
Week 4: building a sense of self	Understanding self-image, self-esteem, and self-compassion. Building self-esteem and self-confidence through self-compassion. Practicing self-love and self-care	Understand how building self-esteem through self-compassion relates to positive outcomes in life and explore strategies to improve our self-esteem and self-confidence
Week 5: protect yourself	Conscious connections and due diligence. Stay safe, substance abuse awareness. It is okay to say no. Staying vigilant on social media	Understand how our connections through friends, peers, and social media influence our thoughts, choices, and decisions in every way
Week 6: prioritize, energize, and recharge yourself	Building self-esteem, optimism, and resilience with mindfulness practices. Practicing the shield of mindfulness. Meditation exercises	Understand what it is like to practice reflection on our thinking with no or as little judgment as possible, allowing us to capture each moment with an awareness of change
Week 7: setting and achieving goals	Discover your passion and purpose. Long-term goals. Short-term goals. What stops us from achieving our goals?	Understand the key elements related to short- and long-term goals and build on setting personal goals for both short term and long term
Week 8: tactics and strategies to achieve goals in life	Definition and meanings of "optimism" and "resilience." Adversity-beliefs-consequences-disputation-energization (ABCDE) model to cope with life adversities. Transforming your self-talk. How to turn adversities into opportunities with accurate choices and decisions	Introduce a model to cope with life adversities, explore how to overcome negative thoughts as well, and turn adversities into opportunities with accurate choices and decisions
Weeks 9 and 10: draft and final project presentations by students		

This study primarily employed a qualitative approach by interviewing the first batch of the pilot phase after successfully completing the S4L program at a mid-Atlantic university in the United States. The semi-structured interviews focused mainly on four sections: (i) the need for such programs, (ii) overall experience during the workshops, (iii) insights into the most impactful sessions or aspects, and (iv) potential areas for improvement and program enhancement.

Additionally, the five-item World Health Organization (WHO) Well-Being Index (WHO-5) [17] and age-appropriate hope scale (State Hope Scale or SHS for this cohort) [18] were administered at three time points: week 0 (baseline), week 8, and week 10 to quantitatively measure the effects of the program in line with the measurements of end points chosen in the protocol [15].

Data analysis

The interview was recorded with the participants' consent, transcribed verbatim, and analyzed using thematic analysis. This method involved coding the data into major themes that corresponded to the interview questions, followed by identifying patterns and trends across the data set. Quotations from the participants were selected to illustrate key points and provide reference to the findings. The results from the anonymized surveys were compared using the average values obtained at various time points.

Ethical considerations

The study was conducted in accordance with ethical guidelines for qualitative research, including obtaining informed consent from all participants, keeping surveys anonymized, and allowing participants to withdraw from the study at any point. Before its commencement, this study, including this qualitative exploration, was reviewed by the Human Subjects Research Committee at Alfred University under US Federalwide Assurance ID IRB00007472, IORG0006213, under the guidelines for the Ethical Treatment of Human Subjects in accordance with Common Rule 45 CFR 46 per the Office for Human Research Protections. It was determined that this research presented no greater than minimal risk to subjects and was given exempt status from all 45 CFR 46 requirements, in accordance with Common Rule 45 CFR 46.104(d)(2) per the Office for Human Research Protections.

## Results

Ten ethnically and gender-diverse undergraduate students were initially randomly approached to participate in the study in the Success4Life program. Of these, six students cited scheduling conflicts with participation in the live sessions, which are essential for the study's core interactions. Four female Caucasian students committed to making themselves available and completed an online consent form. Three were between 18 and 19 years old, and one was 21. After completing the first week of the program, one study participant suggested a fully asynchronous self-study approach using the asynchronous learning management system, complemented by completing instructor-marked journaling assignments. However, she could not prioritize her participation and withdrew from the program. The remaining three participants fully engaged in all weekly online live workshops. This engagement included attending synchronous online lessons, participating in live journaling exercises during these sessions, and submitting their completed journal entries to the LMS after each workshop. Additionally, they had the option to access refresher videos on the LMS. Access to the recorded videos for the following week was automated on the LMS, becoming available only after the instructor marked the journal submissions as complete.

The three participants who completed the study are designated as X, Y, and Z throughout the description of the Results section.

Qualitative group interviews at week 10

Section 1: The Need for Such Programs

When prompted about the need for holistic SEL programs under area 1, Participant X expressed that the program played a crucial role in enhancing self-reflection. The participant noted a significant reduction in stress as a direct outcome of the shifts in their social circle and the emphasis on positive thinking, which facilitated a more fulfilling experience with social interactions.

Participant X said, "I think it would be a really good thing to have this program in our life because I think it draws attention to positive thinking. And really, I know from my experience, throughout this, I've realized who my real friends are and who I should not be really devoting as much time as I have been to. So, a lot of stress has been relieved from me, which has helped me overall, and I've been much happier and more positive."

Both other participants (Participant Y and Participant Z) also recognized the program's role in enhancing self-reflection and reducing negative self-perceptions, which are crucial for mental well-being.

Section 2: Overall Experience During the Workshops

Under Section 2, regarding experience during the eight weeks of the weekly workshops and project discussion, Participant Y highlighted the program's effectiveness in fostering self-awareness and imparting self-care skills that significantly improved personal habits and daily routines.

Participant Y said, "I did not know what self-love was and could not recognize the negative patterns in our life earlier, and now, I understand how to apply these concepts, and I definitely think that I learned that my sleep schedule was really not on track. I've learned how to fix that. I've also been journaling in my spare time."

Participants X and Y shared similar experiences related to self-awareness and self-realization throughout the program.

Section 3: Insights Into the Most Impactful Sessions or Aspects

Under Section 3, on the most helpful aspects, Participant X discussed the profound personal revelations and behavioral changes prompted by the program, emphasizing improvements in self-acceptance and the adoption of healthier habits.

Participant X said, "I found it challenging to really admit to myself where I need to improve and acknowledge what I need to do better. And I think I realized that I do have a lot of negative thoughts about myself at times. So, I learned to reassure myself that I'm doing okay through the lessons that we learned. And I also realized how much time I've been spending on social media. So, I have turned to reading, which I never really had before. So, that was a new thing I started to do because of this. Week 5 and week 6 were a big turning point for me. I really reflected more on myself and what I do each day and how I could change what I'm doing to be better."

Participants Y and Z also emphasized that the week 5 lesson on social media vigilance was highly effective in promoting the mindful use of social media. It helped them avoid mindless scrolling on platforms such as Instagram and TikTok, leading to better sleep at night and increased productivity during the day.

Participant Z reflected on learnings from week 3, especially the "monster voice" exercise for negative hostile self-talk, noting significant progress in transforming their self-talk from negative to positive.

Participant Z said, "If I remember correctly, that's when we did the monster voice stuff. And again, just trying to make myself think not as negatively as I usually would toward myself and trying to keep my inner voice more positive."

Section 4: Potential Areas for Improvement and Program Enhancement

Under Section 4, for feedback for further improvement, overall, the participants appreciated the time allocated for completing journals during the workshops and found it appropriate and beneficial. They also enjoyed the discussions, noting that they were fun and relatable, which enhanced the learning experience. However, Participant X suggested enhancing the program by incorporating more interactive discussions during lessons to facilitate peer learning and deeper engagement.

Participant X said, "I was thinking more like opening up to discussion while the lessons are going on so that we can share our experiences and say we can relate to one another and open up more to get to know one another. Bouncing ideas off each other and relating to one another may be helpful."

Participant Y agreed with the need for a more discussion-oriented format, suggesting that sharing personal experiences related to weekly topics could enhance understanding and empathy among participants.

Participant Y said, "I was also thinking about making it more discussion-based by sharing your own personal experiences. And also maybe, like, each week, throughout the week, come into the lesson with an experience that you had and discuss that and how it could relate to that week's topic with the people so you can understand what each person is going through and how each thing affects someone differently. And I feel like relating it to each other is very beneficial."

**Figure 1:**
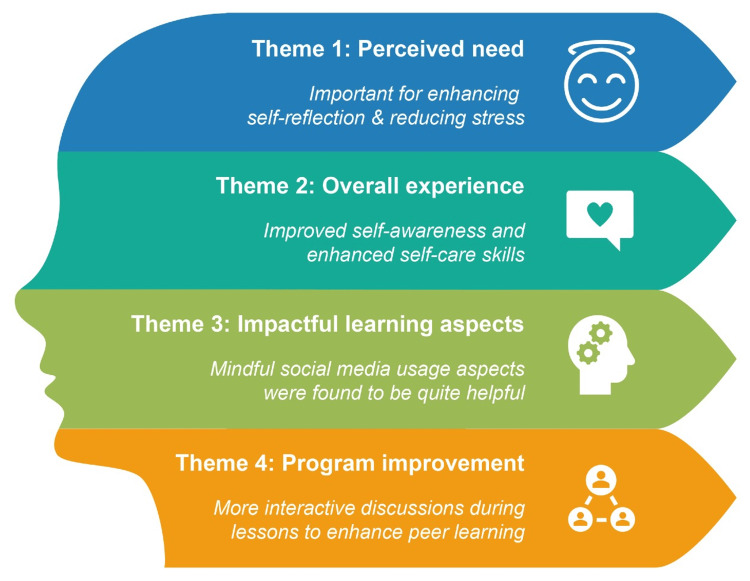
Thematic analysis of qualitative discussion and feedback highlights from the focus group interview. This figure presents a visual summary of the key sections and related themes identified in the focus group discussion with the participants of the Success4Life program. Major themes are linked to main areas to illustrate the connections and flow of insights gathered from the participants' feedback and experiences.

Participant Z pointed to a possible enhancement by shifting to an in-person format to maximize its impact. Figure 1 illustrates the thematic analysis from the focus group interview.

WHO-5 and hope scale survey results

At week 0, the baseline mean WHO-5 score was 58.67. By week 8, the mean WHO-5 score rose to 65.33, and at the conclusion of the program, the mean WHO-5 score reached 68. The mean SHS at baseline was 38.33; it increased slightly at week 8 (38.67) but dropped to the same level as the baseline at week 10 (38.33).

## Discussion

This is the first proof of concept for the effectiveness of a holistic SEL program, S4L, that incorporates the necessary elements of SEL in an intricate curriculum with strategies for goal attainment and the mindful use of social media (Table 1). This qualitative research in the form of feedback evaluation has explored understanding the deeper meaning behind the participants' experiences. Participant X's feedback on the program's effectiveness in fostering positive thinking and re-evaluating social relationships reflects the program's ability to encourage the participants to focus on more supportive and enriching interactions. Participant Y's experience demonstrates the program's ability to impart practical skills, such as improved sleep habits and the adoption of journaling. These changes indicate the program's impact on daily habits and self-care, promoting personal growth and emotional well-being through simple lifestyle modification techniques. In particular, the feedback about understanding the utility of reducing social media usage before bedtime and shifting toward more productive activities such as reading illustrates an essential aspect of lifestyle change through the mindful use of technology and social media facilitated by the program. Our decision to proceed with thematic analysis for a smaller sample size was strategic, allowing for an in-depth exploration of each participant's experience and a more detailed understanding of the impact of the program and feedback on improving its structure and flow for future cohorts of participants. An appropriate sample size estimation is crucial in qualitative studies, as very small sample sizes might be insufficient to claim complete informational redundancy or theoretical saturation. In contrast, larger sample sizes can hinder the intensely focused, case-specific analysis that defines qualitative research. This concept of "information power" is used to better guide the determination of adequate sample sizes in qualitative research. It suggests that the more relevant information a sample holds concerning the study at hand, the fewer participants are required [19]. Determining an appropriate sample size in qualitative studies is fundamentally a judgment call [20]. Empirical evidence suggests that in relatively homogeneous populations (similar to our study population with three Caucasian females who completed the program), data saturation might be achieved with as few as 12 participants, illustrating that smaller sample sizes can fulfill the requirements of comprehensive qualitative analysis [21].

The primary delivery method of the program was virtual synchronous sessions, which were found to be effective. This method facilitated real-time interaction and engagement, aligning with findings from other studies on teaching undergraduate university students, where synchronous learning often leads to higher acceptance, retention, and academic achievement than asynchronous methods [22,23]. Studies have shown that students achieve significantly better results when lectures are delivered synchronously rather than asynchronously. Moreover, overall satisfaction tends to be slightly higher with traditional synchronous lectures, as they encourage more interaction and provide additional support during live sessions [24]. The dropout of a participant from self-directed standalone asynchronous delivery in our study confirms its limitations, particularly in maintaining motivation and engagement without live interactions. This is supported by studies indicating that the cognitive load experienced by students is lower in synchronous settings, potentially due to the immediate feedback and available guidance [25,26]. Our thematic analysis reflected the need for flexibility in educational delivery methods to accommodate different learning preferences among the participants. While virtual synchronous methods proved effective, incorporating face-to-face interactions could enhance engagement for some learners who benefit from in-person connections [27]. Further supporting the need for flexibility in educational delivery methods, a study involving 230 university students examined their perceptions of preferred learning environments in higher education [28]. Overall, two broader perspectives emerged: the need for informal learning environments and the desire for learning settings that allow participation without the necessity of on-campus, face-to-face meetings [28]. These findings shed light on the significance of offering flexible learning options that cater to diverse student needs, including opportunities for virtual and face-to-face interactions. Moreover, improving the structure of synchronous learning components to make them more interactive and engaging is essential for maintaining participant motivation and ensuring comprehensive educational outcomes [29-31].

This study also tracked changes in well-being using the five-item WHO Well-Being Index (WHO-5) across 10 weeks, as they are an integral part of the program's protocol [15]. A difference of 10 or more in the average WHO-5 score is clinically significant [17,32]. The participants' well-being scores increased consistently from the beginning to the end of the program, with nearly a 10-point improvement by the end of 10 weeks. However, due to the small sample size, we cannot establish the statistical significance or confirm the clinical significance of this change. The SHS largely remained at a similar level, which could be attributed to the small sample size or indicate areas for potential enhancement. Although WHO-5 and SHS measure different aspects, WHO-5 has superior psychometric properties and broader applicability, and studies have emphasized the need for improved instruments in hope measurement [17,33,34]. Nevertheless, as suggested by the thematic analysis, incorporating more peer-based discussions and interactive learning activities may help further improve these outcomes in future cohorts. Hence, we have already begun integrating more interactive elements in subsequent cohorts in response to these findings. For example, instead of requiring a real-time completion of journals for each IBL exercise, we now facilitate peer-based real-time discussions among students, allowing them to complete their journals after the workshops. Future studies with these cohorts will determine if this method is more effective than the techniques adopted in this study.

Limitations

While we aimed to recruit a diverse sample by approaching 10 students from various ethnic and gender backgrounds, the participants who ultimately took part were all women and of Caucasian ethnicity. This could influence the generalizability of our findings. We are, however, continuing our efforts to encourage participation from a more diverse group and confirm the results across different demographic populations.

## Conclusions

In conclusion, our preliminary findings reflect positively on the effectiveness of the S4L program, particularly its virtual synchronous sessions. The areas for improvement by incorporating more peer-based learning and interactivity are essential for enhancing the program's design and ensuring it meets the diverse needs of the participants effectively.

## References

[1] Dussault M, Thompson RB (2024). Fundamental themes in social-emotional learning: a theoretical framework for inclusivity. Int J Environ Res Public Health.

[2] Shi J, Cheung AC (2024). Effective components of social emotional learning programs: a meta-analysis. J Youth Adolesc.

[3] Posamentier J, Seibel K, DyTang N (2023). Preventing youth suicide: a review of school-based practices and how social-emotional learning fits into comprehensive efforts. Trauma Violence Abuse.

[4] Takizawa Y, Matsumoto Y, Ishimoto Y (2024). Effectiveness of universal social-emotional learning programs for Japanese higher education students: a meta-analytic review. Health Open Res.

[5] Dowling K, Barry MM (2020). The effects of implementation quality of a school-based social and emotional well-being program on students’ outcomes. Eur J Investig Health Psychol Educ.

[6] Castaldelli-Maia JM, Matakas NK (2024). Could school programs based on social-emotional learning prevent substance abuse among adolescents?. World J Psychiatry.

[7] Setia S, Tichy M, Gilbert F (2024). Innovating social-emotional learning to enhance positive engagement of youth with social media: a comprehensive review of why and how. Cureus.

[8] Cullen J, Muntz A, Marsh S, Simmonds L, Mayes J, O'Neill K, Duncan S (2024). Impacts of digital technologies on child and adolescent health: recommendations for safer screen use in educational settings. N Z Med J.

[9] Wang Q, Qin Z, Xing X, Zhu H, Jia Z (2024). Prevalence of cannabis use around the world: a systematic review and meta-analysis, 2000-2024. China CDC Wkly.

[10] Degenhardt L, Webb P, Colledge-Frisby S (2023). Epidemiology of injecting drug use, prevalence of injecting-related harm, and exposure to behavioural and environmental risks among people who inject drugs: a systematic review. Lancet Glob Health.

[11] Sharma MK, John N, Sahu M (2020). Influence of social media on mental health: a systematic review. Curr Opin Psychiatry.

[12] Andersen SL (2019). Stress, sensitive periods, and substance abuse. Neurobiol Stress.

[13] Behrendt S, Wittchen HU, Höfler M, Lieb R, Beesdo K (2009). Transitions from first substance use to substance use disorders in adolescence: is early onset associated with a rapid escalation?. Drug Alcohol Depend.

[14] Setia S, Krägeloh C, Bandyopadhyay G, Subramaniam K (2021). Inculcating dispositional optimism for prevention of mental and substance use disorders throughout and after the coronavirus disease-19 pandemic. Altern Complement Therap.

[15] Setia S, Furtner D, Bendahmane M, Tichy M (2022). Success4life youth empowerment for promoting well-being and boosting mental health: protocol for an experimental study. JMIR Res Protoc.

[16] Palinkas LA (2014). Qualitative and mixed methods in mental health services and implementation research. J Clin Child Adolesc Psychol.

[17] Topp CW, Østergaard SD, Søndergaard S, Bech P (2015). The WHO-5 Well-Being Index: a systematic review of the literature. Psychother Psychosom.

[18] Edwards LM, Rand KL, Lopez SJ, Snyder CR (2007). Understanding hope: a review of measurement and construct validity research. Oxford handbook of methods in positive psychology.

[19] Malterud K, Siersma VD, Guassora AD (2016). Sample size in qualitative interview studies: guided by information power. Qual Health Res.

[20] Sandelowski M (1995). Sample size in qualitative research. Res Nurs Health.

[21] Boddy CR (2016). Sample size for qualitative research. Qual Mark Res.

[22] Khalil R, Mansour AE, Fadda WA (2020). The sudden transition to synchronized online learning during the COVID-19 pandemic in Saudi Arabia: a qualitative study exploring medical students' perspectives. BMC Med Educ.

[23] Yadav SK, Para S, Singh G, Gupta R, Sarin N, Singh S (2021). Comparison of asynchronous and synchronous methods of online teaching for students of medical laboratory technology course: a cross-sectional analysis. J Educ Health Promot.

[24] Fabriz S, Mendzheritskaya J, Stehle S (2021). Impact of synchronous and asynchronous settings of online teaching and learning in higher education on students’ learning experience during COVID-19. Front Psychol.

[25] Hung CT, Wu SE, Chen YH, Soong CY, Chiang CP, Wang WM (2024). The evaluation of synchronous and asynchronous online learning: student experience, learning outcomes, and cognitive load. BMC Med Educ.

[26] Zhu E (2006). Interaction and cognitive engagement: an analysis of four asynchronous online discussions. Instr Sci.

[27] Photopoulos P, Tsonos C, Stavrakas I, Triantis D (2023). Remote and in-person learning: utility versus social experience. SN Comput Sci.

[28] Valtonen T, Leppänen U, Hyypiä M (2021). Learning environments preferred by university students: a shift toward informal and flexible learning environments. Learn Environ Res.

[29] Ichikura K, Watanabe K, Moriya R (2024). Online vs. face-to-face interactive communication education using video materials among healthcare college students: a pilot non-randomized controlled study. BMC Med Educ.

[30] Darling-Hammond L, Flook L, Cook-Harvey C, Barron B, Osher D (2020). Implications for educational practice of the science of learning and development. Appl Dev Sci.

[31] Falcon RM, Alcazar RM, Capistrano NJ (2024). Interactive journal club: a learning method to enhance collaboration and participation among medical students. Acta Med Philipp.

[32] Allgaier AK, Pietsch K, Frühe B, Prast E, Sigl-Glöckner J, Schulte-Körne G (2012). Depression in pediatric care: is the WHO-Five Well-Being Index a valid screening instrument for children and adolescents?. Gen Hosp Psychiatry.

[33] Quansah F, Hagan JE Jr, Ankomah F, Agormedah EK, Nugba RM, Srem-Sai M, Schack T (2022). Validation of the WHO-5 Well-Being Scale among adolescents in Ghana: evidence-based assessment of the internal and external structure of the measure. Children (Basel).

[34] Redlich-Amirav D, Ansell LJ, Harrison M, Norrena KL, Armijo-Olivo S (2018). Psychometric properties of hope scales: a systematic review. Int J Clin Pract.

